# Magnetic Sphincter Augmentation Outcomes in Severe Gastroesophageal Reflux Disease

**DOI:** 10.3389/fmed.2021.645592

**Published:** 2021-11-02

**Authors:** Davide Ferrari, Stefano Siboni, Carlo Galdino Riva, Guglielmo Guerrazzi, Andrea Lovece, Luigi Bonavina

**Affiliations:** Division of General and Foregut Surgery, Department of Biomedical Sciences for Health, Istituto di Ricovero e Cura a Carattere Scientifico (IRCCS) Policlinico San Donato, University of Milan, Milan, Italy

**Keywords:** gastroesophageal reflux disease, hiatus hernia, esophagitis, Barrett's esophagus, DeMeester score, magnetic sphincter augmentation

## Abstract

**Introduction:** Outcomes of laparoscopic procedures for gastroesophageal reflux disease (GERD) are variable depending on surgical expertise and/or patient-related factors. Some procedures may be inadequate in patients with severe disease. Effectiveness of laparoscopic magnetic sphincter augmentation (MSA) has not been extensively tested in patients with severe disease.

**Methods:** A prospectively collected database was analyzed to identify patients who underwent MSA at a single institution. Individuals who had previous esophago-gastric surgery were excluded. Severe GERD was defined as lower esophageal sphincter pressure <5 mmHg, distal esophageal amplitude <30 mmHg, Barrett's metaplasia, stricture or grade C-D esophagitis, and/or DeMeester score >50. Clinical characteristics and outcomes of patients with severe GERD were compared with those of patients with mild to moderate GERD who served as control group.

**Results:** Over the study period, a total of 336 patients met the inclusion criteria, and 102 (30.4%) had severe GERD. The median follow-up was 24 months (IQR = 75) in severe GERD patients and 32 months (IQR = 84) in those with non-severe GERD. Patients with severe GERD had a higher rate of dysphagia and higher GERD-HRQL scores. After the MSA procedure, symptoms, health-related quality of life scores, and proton-pump inhibitors consumption significantly decreased in both groups (*p* < 0.05). No difference between groups was found in the prevalence of severe post-operative dysphagia, the need for endoscopic dilation or device removal, and the DeMeester score.

**Conclusion:** Laparoscopic MSA is safe and effective in reducing symptoms, PPI use, and esophageal acid exposure also in patients with severe GERD.

## Introduction

The pooled prevalence of gastro-esophageal reflux disease (GERD) is 14%, with more than 1 billion of individuals affected and an enormous economic burden on health-care systems around the world (1). The novel laparoscopic magnetic sphincter augmentation (MSA) procedure was developed to offer a minimally invasive and standardized alternative to the total (360°) Nissen and the partial (270°) Toupet fundoplication. Both total and partial fundoplication still represent the surgical standard for GERD patients who are refractory to proton-pump inhibitors (PPI) therapy, but these procedures are widely underused due to lack of reproducibility and broad variability in outcomes (2–4). Magnetic sphincter augmentation has proven safe and effective in reducing GERD symptoms, consumption of PPI, and esophageal acid exposure for up to 12 years of follow-up (5). Initially, MSA was mainly performed in patients with mild to moderate GERD presenting with no or minimal anatomical alterations and esophagitis grade B or less (6). Throughout the years, inclusion criteria have been expanded to include patients with hiatal hernia >3 cm, esophagitis >grade B, and Barrett's esophagus, but only a few studies have evaluated the outcomes of MSA in patients with severe GERD (7–11).

Pre-operative indicators of GERD severity have previously been defined based on manometric, pH-monitoring, and endoscopic findings that may predict failure of a partial fundoplication. Of note, the pre-operative DeMeester pH score showed 86% sensitivity for predicting surgical failure (12). Aim of this study was to evaluate the short- and long-term effectiveness of MSA in patients with severe GERD compared with individuals with mild to moderate disease.

## Materials and Methods

A retrospective analysis was conducted using a prospectively collected database of patients who had undergone MSA implantation. The study protocol was approved by the Institutional Review Board. Inclusion criteria were age between 18 and 65 years and a minimum post-operative follow-up of 6 months. Exclusion criteria were previous esophagogastric surgery and documented allergy to titanium or nickel. Patients were included in the severe GERD group if one or more of the following conditions were present pre-operatively: LES basal pressure <5 mmHg or distal esophageal amplitude <30 mmHg on esophageal manometry, biopsy-proven Barrett's metaplasia, presence of stricture or grade C-D esophagitis on upper gastrointestinal endoscopy, and DeMeester score >50 on ambulatory esophageal pH monitoring (12). The remaining patients were included in the mild to moderate GERD group. Pre- and post-operative patient characteristics of the two patient groups were compared.

### Pre-operative Assessment

All patients underwent clinical assessment by completing the gastroesophageal reflux disease health-related quality of life (GERD-HRQL) questionnaire on-PPI. The GERD-HRQL score is based on 10 questions and all queries have a score ranging from 0 to 5. A GERD-HRQL score >15 is considered abnormal (13).

All patients also underwent a full diagnostic assessment including barium swallow study, endoscopy, esophageal pH-monitoring or pH-impedance off PPI, and esophageal manometry. Upper gastrointestinal endoscopy was performed to assess the presence of esophagitis according to the Los Angeles classification, biopsy-proven Barrett's esophagus, peptic stricture, or hiatus hernia. An esophageal pH or pH-impedance study was performed using a trans-nasal catheter or a wireless system (BRAVO™), and the DeMeester score and esophageal acid exposure time were collected. Standard or high-resolution esophageal manometry investigated Lower Esophageal Sphincter (LES) resting pressure, LES overall and abdominal length, and distal esophageal amplitude.

### Surgical Technique

The laparoscopic MSA implantation was performed under general anesthesia, as previously described (14). The gastroesophageal junction is dissected, the posterior vagus nerve is identified and separated from the esophageal wall, and the esophagus is encircled with a Penrose drain. No short gastric vessels are divided. In patients with hiatal hernia ≥3 cm, mediastinal dissection and posterior crural repair are routinely performed. The esophageal circumference is measured with a magnetic sizer device. The correct size of MSA is decided by increasing 2 or 3 beads from the point of sizer release. Finally, the MSA device (Linx Reflux Management System, Ethicon, Johnson & Johnson, Shoreview, Mn, USA) is inserted through the retroesophageal tunnel and locked anteriorly.

### Post-operative Follow-Up

Patients underwent post-operative clinical assessment with GERD-HRQL and functional outcome swallowing scale (FOSS) questionnaire (15) to analyze reflux symptoms, quality of life, and dysphagia at 2 weeks, 6 months, and then each year after the operation. A FOSS score >1 identified severe post-operative dysphagia. Upper gastrointestinal endoscopy, barium swallow study, esophageal manometry, and esophageal pH monitoring were performed between 6 and 12 months after surgery and repeated thereafter according to specific clinical circumstances or as a part of investigational studies.

### Statistical Analysis

Continuous variables are reported as mean ± standard deviation (SD) or median with interquartile range (IQR) when appropriate. Variables were compared between patients with mild to moderate GERD disease to patients with severe GERD, as defined before. Statistical analysis was performed through Wilcoxon test, Mann–Whitney *U*, Student's t test and Chi-square test as appropriate. When pre- and post-operative variables were compared, a two-tailed paired Student's t test was used. A *p* < 0.05 was considered statistically significant. Statistical analysis was performed using SPSS software 23.0 (IBM, Armonk, New York, U.S.).

## Results

**Figure 1:**
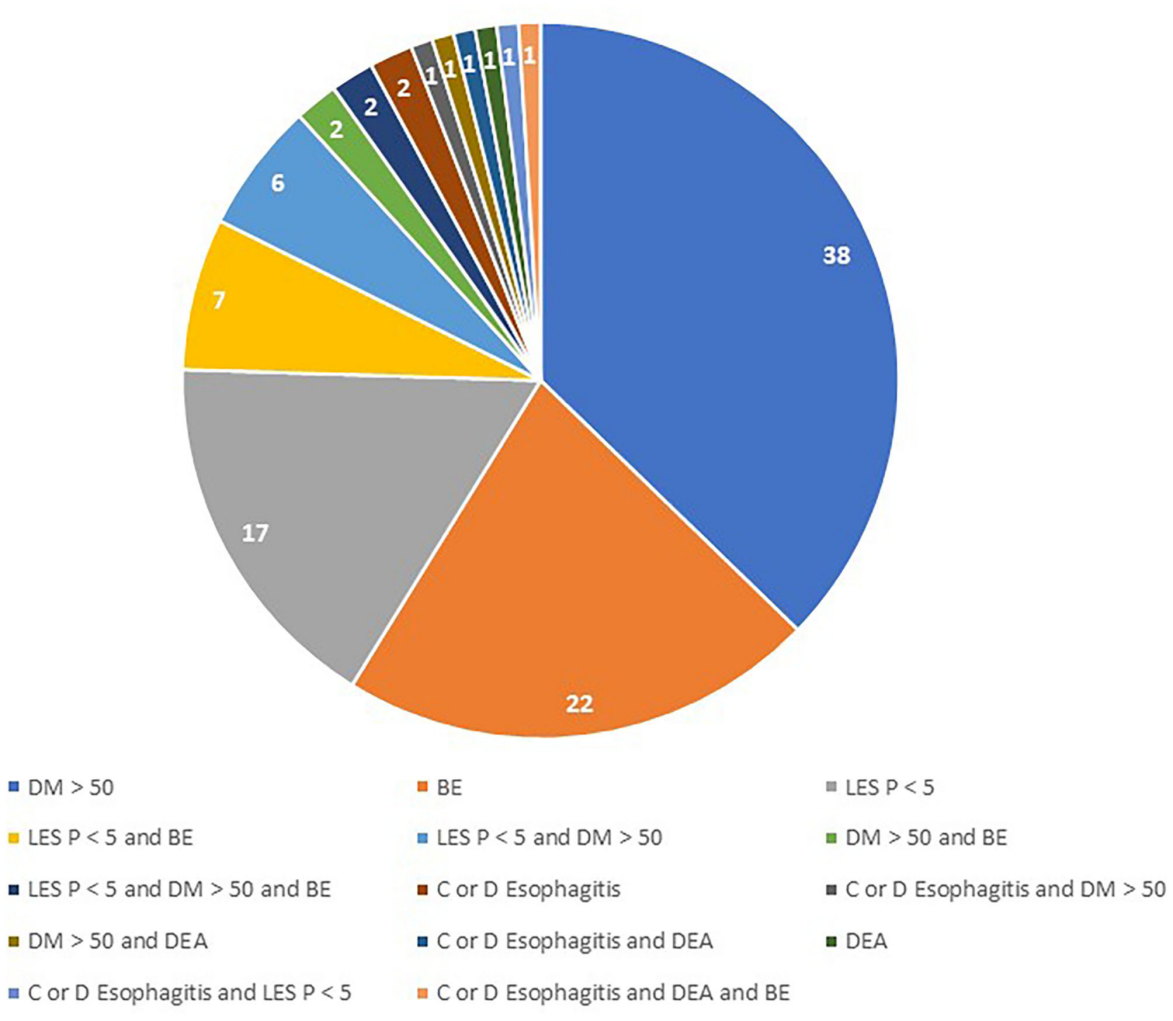
Combination of pre-operative abnormalities in patients with severe GERD. DM, DeMeester Score; BE, Barrett's Esophagus; LES P, LES residual pressure; DEA, Distal esophageal aperistalsis.

A total of 336 (32.7% female) patients were included in the study. Among them, 102 patients (30.4%) met the criteria for inclusion in the severe GERD group. The main pre-operative reasons accounting for disease severity were DeMeester score >50 (49% of patients), biopsy-proven Barrett's metaplasia (33.3%), and LES basal pressure <5 mmHg (27.5%; Table 1). A total of 19 patients met two inclusion criteria, and three patients met three inclusion criteria. There were no patients with peptic esophageal strictures found at pre-operative endoscopy (Figure 1).

**Table 1:** Distribution of pre-operative abnormalities in patients with severe GERD.

	***N* = 102 (%)**
LES pressure <5 mmHg	33 (32.4)
Distal esophageal amplitude <30 mmHg	4 (3.9)
Biopsy-proven Barrett's metaplasia	34 (33.3)
Presence of a stricture	0 (0)
Grade C or D esophagitis on endoscopy	6 (5.8)
DeMeester score > 50	50 (49)

Demographic characteristics were similar in the two patient groups. However, patients with severe GERD had a higher rate of pre-operative dysphagia, higher scores of GERD-HRQL questionnaire, and higher PPI use (Table 2).

**Table 2:** Baseline demographic and clinical data of patients with severe or non-severe GERD.

	**Non-severe GERD** **(*n* = 234)**	**Severe GERD** **(*n* = 102)**	***p***
Age, years	45.2 (±13.8)	46.2 (±13.3)	0.5374
Female, *n* (%)	72 (30.7)	38 (37.2)	0.2435
Body mass index, kg/m^2^	24.8 (±3.7)	25.3 (±3.9)	0.2634
Heartburn, *n* (%)	192 (82.1)	81 (79.4)	0.5602
Regurgitation, *n* (%)	125 (53.4)	53 (52)	0.8134
Dysphagia, *n* (%)	13 (5.5)	12 (11.7)	0.0460
Symptom duration (years)	8.3 (±6.9)	9.7 (±5.1)	0.0665
PPI use, *n* (%)	167 (71.3)	88 (86.2)	0.0034
PPI therapy (years)	6.7 (±5.6)	7.1 (±5.1)	0.5369
GERD-HRQL score	19.2 (±7.7)	21.0 (±7.5)	0.0479
Atypical symptoms, *n* (%)	6 (2.6)	3 (2.9)	0.8760

*Continuous variables are expressed using mean values (±SD)*.

Table 3 shows that application of the study inclusion predicted disease severity, i.e., patients with severe GERD had lower LES resting pressure, weaker peristaltic amplitude, and greater esophageal acid exposure compared to patients with mild disease. There were no significant differences regarding the intraoperative variables, except for post-operative length of hospital stay (Table 4).

**Table 3:** Baseline pre-operative findings in patients with severe or non severe GERD.

	**Non-severe GERD** **(*n* = 234)**	**Severe GERD** **(*n* = 102)**	***p***
Hiatal hernia, *n* (%)	177 (75.6)	84 (82.3)	0.1760
Hiatal hernia, cm	1.7 (±1.3)	1.9 (±1.4)	0.2062
LES resting pressure, mmHg	18.8 (±11.2)	13.4 (±11.8)	0.0001
LES overall length, cm	2.9 (±1.5)	2.8 (±1.4)	0.5669
LES abdominal length, cm	1.2 (±1.3)	1.1 (±1.3)	0.5172
DEA, mmHg	74.2 (±35.4)	62.1 (±23.8)	0.0018
Total acid exposure time, %	6.5 (±3.7)	13.3 (±9.3)	<0.0001
DeMeester score	26.2 (±12)	58.3 (±33.5)	<0.0001

*Continuous variables are expressed using mean values (±SD)*.

**Table 4:** Intraoperative and clinical course of patients with severe or non-severe GERD.

	**Non-severe GERD** **(*n* = 234)**	**Severe GERD** **(*n* = 102)**	***p***
Duration of intervention, min	61.4 (30)	61.1 (24.5)	0.9292
Number of beads	13.9 (1.3)	14.1 (1.4)	0.2062
Crural repair, *n* (%)	94 (40.2)	50 (49)	0.1345
Length hospital stay, days	1.4 (0.7)	1.2 (0.6)	0.0125

*Continuous variables are expressed using mean values (SD)*.

**Figure 2:**
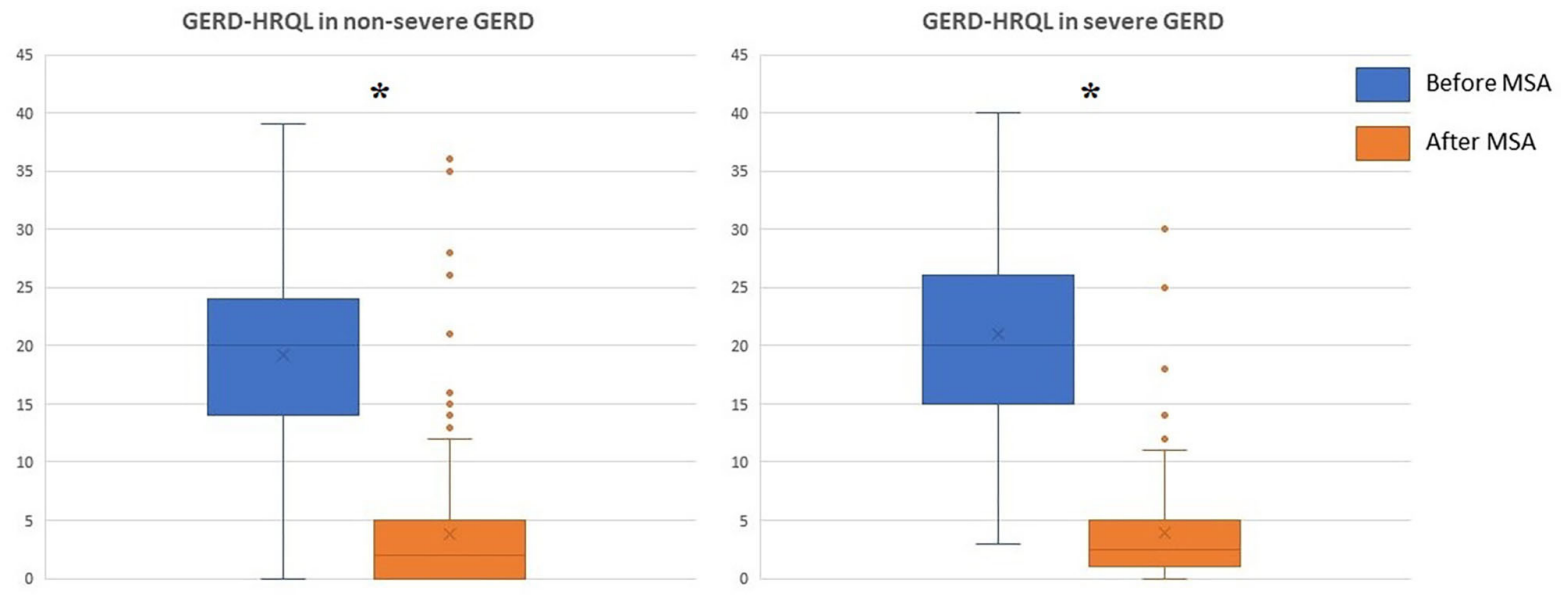
GERD-HRQL scores before and after laparoscopic MSA in patients with or without severe GERD. GERD-HRQL, gastroesophageal reflux disease health-related quality of life; MSA, magnetic sphincter augmentation; GERD, gastro-esophageal reflux disease; *p < 0.05.

**Figure 3:**
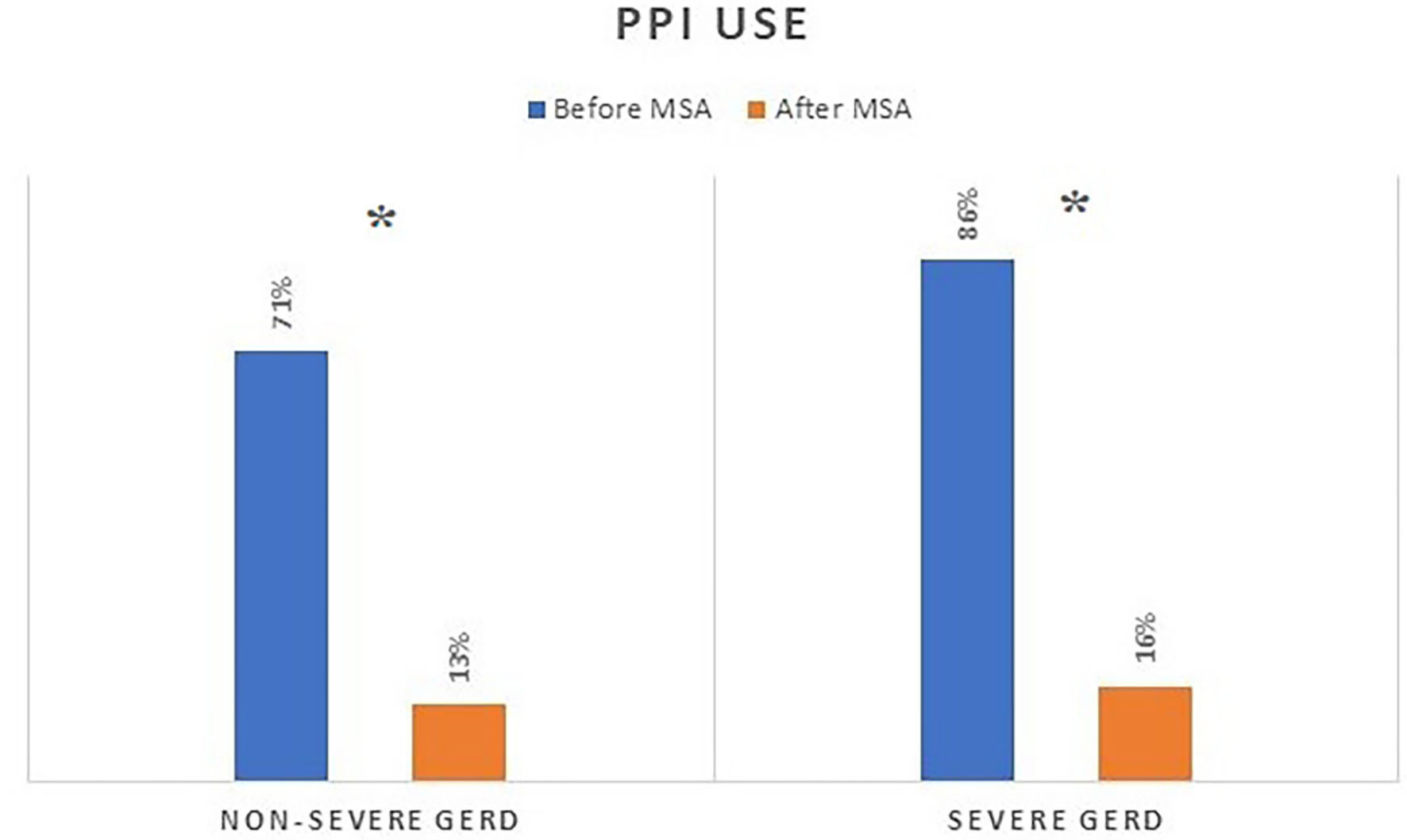
PPI consumption before and after MSA procedure in patients with or without severe GERD. PPI, proton-pump inhibitors; MSA, magnetic sphincter augmentation; GERD, gastro-esophageal reflux disease; *p < 0.05.

**Figure 4:**
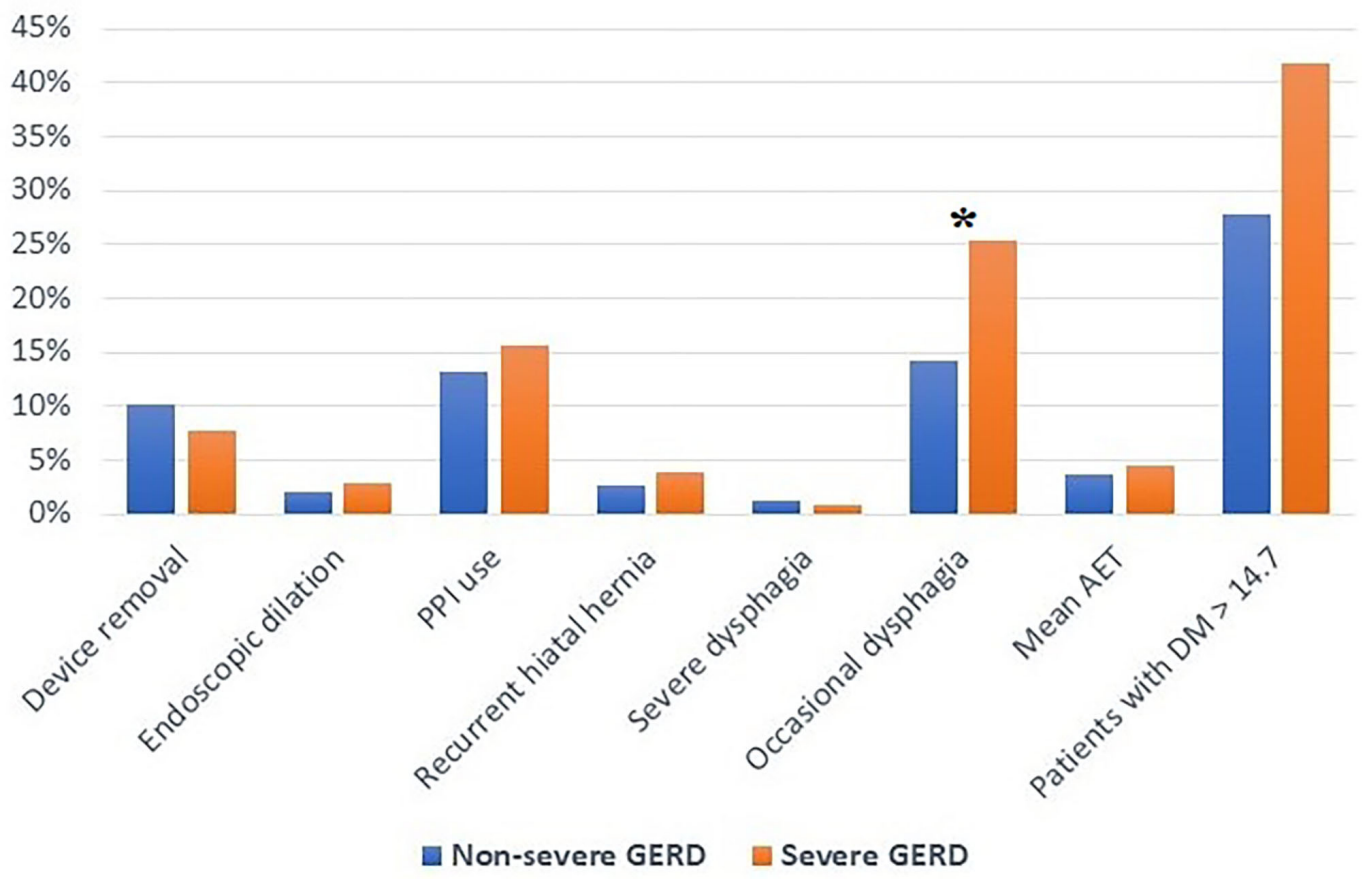
Post-operative outcomes in patients with severe or non severe GERD. PPI, proton-pump inhibitors; AET, acid exposure time; DM, DeMeester score; GERD, gastro-esophageal reflux disease; *p < 0.05.

**Figure 5:**
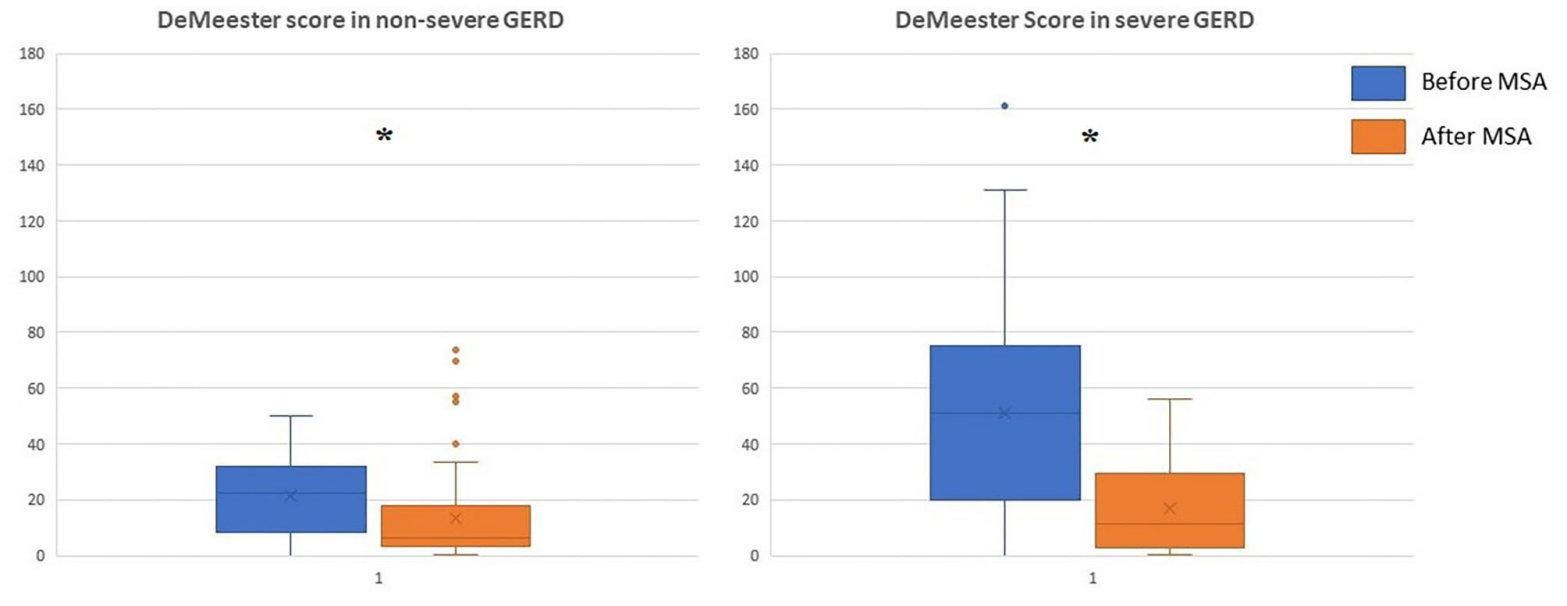
DeMeester score in patients with severe or non severe GERD before and after MSA procedure. MSA, magnetic sphincter augmentation; GERD, gastro-esophageal reflux disease; *p < 0.05.

The median follow-up was 24 months (IQR = 75) and 32 months (IQR = 84) in the severe and in the non-severe GERD group, respectively. In both groups, GERD-HRQL scores and use of PPI significantly decreased compared to baseline (*p* < 0.05; Figures 2, 3). Post-operative outcomes and complications are shown in Table 5. Patients with severe GERD had a higher rate of occasional post-operative dysphagia (25.4 vs. 14.1%, *p* = 0.0124), but less individuals required device removal (8 vs. 24 patients, *p* = NS; Figure 4). Overall, 122 patients underwent esophageal manometry at a median of 12 months (IQR = 30) after surgery. The LES resting pressure significantly increased in both groups, but DEA increased more consistently and reached statistical significance only in patients with non-severe GERD. A total of 108 patients underwent post-operative esophageal pH-monitoring at a median of 28 months (IQR = 51) after surgery, 72 (30.7%) in the non severe GERD group, and 36 (35.5%) in the severe GERD group (*p* = 0.3871). No significant differences were found between the groups in terms of acid exposure time, DeMeester score, or number of patients with DeMeester score >14.7. However, there was a trend toward higher acid exposure in patients with pre-operative severe GERD (Figure 5).

**Table 5:** Post-operative outcomes in patients undergoing MSA for severe or non-severe GERD.

	**Non-severe GERD** **(*n* = 234)**	**Severe GERD** **(*n* = 102)**	***p***
Follow-up, months	50.8 (±44.2)	49.6 (±43.7)	0.8185
GERD-HRQL score	3.8 (±5.7)	3.9 (±4.8)	0.8770
Use of PPI, *n* (%)	31 (13.2)	16 (15.6)	0.5597
Occasional post-operative dysphagia, *n* (%)	33 (14.1)	26 (25.4)	0.0124
Recurrent hiatal hernia, *n* (%)	6 (2.6)	4 (3.9)	0.5209
Endoscopic dilation, *n* (%)	5 (2.1)	3 (2.9)	0.6562
Device removal, *n* (%)	24 (10.2)	8 (7.8)	0.4903
LES resting pressure, mmHg^δ^	24.3 (± 10.4)	21.4 (±12.3)	0.0271
LES overall length, cm^δ^	3.2 (±1.3)	3.1 (±1.4)	0.5270
LES abdominal length, cm^δ^	1.4 (±1.4)	1.4 (±1.5)	1.0000
DEA, mmHg^δ^	82.4 (±44.4)	66.6 (±28.9)	0.0011
Acid exposure time, %^*^	3.6 (±4.4)	4.5 (±4.4)	0.0856
DeMeester score^*^	13.4 (±15.9)	17 (±16.3)	0.0591
DeMeester >14.7, *n* (%)	20 (27.8)	15 (41.7)	0.1476

*Continuous variables are expressed using mean values (±SD)*.

δ *122 patients underwent post-operative esophageal manometry, 40 in the severe GERD group*.

* *108 patients underwent post-operative pH study, 36 in the severe GERD group*.

## Discussion

This observational study shows that MSA is a safe and effective procedure in patients presenting with severe GERD and that clinical outcomes are similar to those observed in patients with mild to moderate disease (16). GERD is a spectrum disease presenting with different phenotypes. The goal of predicting surgical outcomes based on the presence of erosive esophagitis has largely failed due to the difficulties in precisely recognizing the mucosal phenotype. In fact, most patients are treated with high-dose PPI which can masquerade the presence of erosive esophagitis. Finally, given the lack of a universally recognized definition of disease severity, using a more composite definition of severe GERD may in part resolve this problem and help in identifying patients at greater risk of progressive disease (17–19).

The goal of antireflux surgery is to restore competence of the esophagogastric junction, but laparoscopic Nissen and Toupet fundoplication carry the burden of post-operative side effects and high variability in outcomes (20–22). Horvath et al. described six independent measures of disease severity associated to surgical failure after laparoscopic Toupet fundoplication. Interestingly a pre-operative DeMeester score >50 was 86% sensitive for predicting surgical failure (12). More recently, Schwameis et al. (23) have stratified 334 patients undergoing MSA using the previous criteria. They found that MSA is an effective therapeutic option for patients with severe GERD as defined by a pre-operative DeMeester score ≥50. Both patients' groups significantly improved at a mean follow-up of 13.6 months, but among patients with severe GERD the rate of distal esophageal acid exposure normalization tended to be lower (*p* = 0.109) and more patients were using PPI (*p* < 0.041).

In the present study, patients with severe GERD undergoing MSA had excellent outcomes at a mean of 50 months of follow-up, with a significant improvement of GERD-HRQL scores and decreased PPI use compared to baseline. These results are similar to what we observed in our cohort of control patients with mild to moderate GERD. Interestingly, occasional dysphagia was more common in the severe GERD group, both in the pre- and in the post-operative period. This higher prevalence of pre-operative dysphagia may be explained by a motility dysfunction induced by long-standing reflux or by an occasional diaphragmatic entrapment of a sliding hiatus hernia (24, 25). On the other hand, the higher rate of crural repair associated with MSA implantation in patients with severe GERD may explain the significantly higher rate of post-operative dysphagia observed in these individuals. In fact, crural repair has an additive effect on LES augmentation, and the combination of hiatoplasty and MSA may increase the incidence of post-operative dysphagia (26, 27). Last but not least, there were no significant differences between the two patient groups regarding the post-operative rates of recurrent hiatal hernia, endoscopic dilation, and laparoscopic device removal. The most common reasons for explant were persistent heartburn/regurgitation (3.6%), dysphagia (1.8%), and erosion (1.8%). There was no significant morbidity or mortality associated with these revisional laparoscopic procedures (5, 28).

Furthermore, a significant reduction in the DeMeester score compared to baseline was noted in both patient groups. Although there was a trend toward an abnormally higher post-operative score in the severe GERD group, the majority of these patients reached pH normalization. Despite the fact that even patients with severe GERD may benefit from MSA, we hypothesize that an earlier surgical intervention has the potential to prevent anatomical deterioration of the esophago-gastric barrier and Barrett's mucosal changes (26). In two recent studies, age younger than 40-45 years, male sex, GERD-HRQL total score >15, and abnormal DeMeester score were independent predictors of favorable outcome after MSA (5, 27).

The retrospective design of this research, the possible selection bias, and the lack of systematic post-operative ambulatory pH studies represent the main study limitations. Another intrinsic study limitation is the lack of a universally recognized definition of severe GERD.

In conclusion, the laparoscopic MSA procedure can safely be offered to patients with GERD regardless of the severity of the disease as assessed by a set of anatomic and physiologic indicators. However, further prospective studies with longer follow-up are needed and special caution is needed in individuals with long-standing Barrett's esophagus because of the risk of progression to dysplasia and cancer in spite of adequate reflux control.

## Data Availability Statement

The raw data supporting the conclusions of this article will be made available by the authors, without undue reservation.

## Ethics Statement

Ethical review and approval was not required for the study on human participants in accordance with the local legislation and institutional requirements. The patients/participants provided their written informed consent to participate in this study.

## Author Contributions

DF and LB designed the study, analyzed the data, and were major contributors in writing and reviewing the manuscript. SS, CR, AL, and GG searched the literature, made the statistical analysis, and reviewed the manuscript. All authors contributed to the article and approved the submitted version.

## Funding

This study was supported by A.I.R.ES. (Associazione Italiana Ricerca ESofago).

## Conflict of Interest

The authors declare that the research was conducted in the absence of any commercial or financial relationships that could be construed as a potential conflict of interest.

## Publisher's Note

All claims expressed in this article are solely those of the authors and do not necessarily represent those of their affiliated organizations, or those of the publisher, the editors and the reviewers. Any product that may be evaluated in this article, or claim that may be made by its manufacturer, is not guaranteed or endorsed by the publisher.
